# Public Perceptions About Monkeypox on Twitter: Thematic Analysis

**DOI:** 10.2196/48710

**Published:** 2023-11-03

**Authors:** Abimbola Leslie, Omolola Okunromade, Abeed Sarker

**Affiliations:** 1 Department of Radiology Robert Larner College of Medicine University of Vermont Burlington, VT United States; 2 Department of Health Policy and Community Health Jiann-Ping Hsu College of Public Health Georgia Southern University Atlanta, GA United States; 3 Department of Biomedical Informatics School of Medicine Emory University Atlanta, GA United States

**Keywords:** monkeypox, social media, public health, Twitter, perception, digital platform, infectious disease, outbreak, awareness, analyses, misinformation

## Abstract

**Background:**

Social media has emerged as an important source of information generated by large segments of the population, which can be particularly valuable during infectious disease outbreaks. The recent outbreak of monkeypox led to an increase in discussions about the topic on social media, thus presenting the opportunity to conduct studies based on the generated data.

**Objective:**

By analyzing posts from Twitter (subsequently rebranded X), we aimed to identify the topics of public discourse as well as knowledge and opinions about the monkeypox virus during the 2022 outbreak.

**Methods:**

We collected data from Twitter focusing on English-language posts containing key phrases like “monkeypox,” “mpoxvirus,” and “monkey pox,” as well as their hashtag equivalents from August to October 2022. We preprocessed the data using natural language processing to remove duplicates and filter out noise. We then selected a random sample from the collected posts. Three annotators reviewed a sample of the posts and created a guideline for coding based on discussion. Finally, the annotators analyzed, coded, and manually categorized them first into topics and then into coarse-grained themes. Disagreements were resolved via discussion among all authors.

**Results:**

A total of 128,615 posts were collected over a 3-month period, and 200 tweets were selected and included for manual analyses. The following 8 themes were generated from the Twitter posts: monkeypox doubts, media, monkeypox transmission, effect of monkeypox, knowledge of monkeypox, politics, monkeypox vaccine, and general comments. The most common themes from our study were monkeypox doubts and media, each accounting for 22% (44/200) of the posts. The posts represented a mix of useful information reflecting emerging knowledge on the topic as well as misinformation.

**Conclusions:**

Social networks, such as Twitter, are useful sources of information in the early stages of outbreaks. Close to real-time identification and analyses of misinformation may help authorities take the necessary steps in a timely manner.

## Introduction

The monkeypox virus is a zoonotic orthopoxvirus similar to the virus that causes smallpox. The first known outbreak of this virus among humans was reported in 1970 in a sub-Saharan region, specifically the Democratic Republic of the Congo [1]. Monkeypox infection in humans has mostly been reported in Africa, typically originating from contact with wildlife reservoirs [2]. Countries like Benin, Cameroon, the Central African Republic, the Democratic Republic of the Congo, Gabon, Cote d’Ivoire, Liberia, Nigeria, the Republic of the Congo, Sierra Leone, and South Sudan have reported cases of monkeypox [3]. Monkeypox has emerged as one of the most important orthopoxviruses for public health, with a case fatality ratio of 3%-6% [4]. In 2022, monkeypox infections were reported in more than 50 countries, including the United States and multiple European countries [5] Consequently, the World Health Organization declared that the risk of monkeypox at the global level was “moderate” [5] and later declared it a “public health emergency of international concern” [6].

Although monkeypox has been endemic in parts of Africa for decades, prior to 2022, there had been relatively limited research on the topic compared to other infectious diseases, such as COVID-19 [2]. The 2022 outbreak, however, ignited a flurry of research. According to the US Centers for Disease Control and Prevention, as of March 16, 2023, there were 86,500 confirmed cases of monkeypox globally [7]. The vast majority of these confirmed cases (n=30,262) were in the United States, and more than 85,000 cases were from locations that had not historically reported monkeypox. Consequently, the topic of monkeypox received considerable attention on social media (eg, Twitter) [8]. The COVID-19 pandemic, which preceded the global monkeypox outbreak, was the first pandemic of its scale in the social media era, and it helped bring to light the power of social media at times of public health crises. For example, studies have shown that social media data could be leveraged to detect COVID-19 outbreaks in close to real time, detect symptoms, conduct geolocation-specific syndromic surveillance, and study public perceptions and mental health statuses. At the same time, social media has emerged as a platform that is exploited to spread misinformation and market fraudulent products. Although social media plays both positive and negative roles at times of pandemics and disease outbreaks in the modern world, what is important is that the knowledge generated over such platforms is studied to better understand public perceptions and characterize communications. In the context of monkeypox, it is of paramount importance to understand people’s perceptions and knowledge of the topic. Specifically, as the COVID-19 pandemic has demonstrated, public health policies and government actions regarding infectious disease outbreaks need to take into account the information circulating on public social media so as to maximize their impacts. It is also important to detect potential misinformation early so that its negative impacts are mitigated.

Based on these motivations, in this paper, we present a manual, thematic analysis of Twitter posts discussing monkeypox. Our analysis reveals the key themes in monkeypox-related chatter on Twitter and their relative distributions. This study adds to the body of evolving knowledge about monkeypox, particularly knowledge crowdsourced from social media subscribers.

## Methods

### Ethical Considerations

This is a manual mixed methods descriptive study to characterize public tweets mentioning **“**monkeypox.**”** This study was deemed exempt from detailed review by the Emory University institutional review board (category 4; publicly available data). Informed consent was not obtained for our study because our data were publicly available at the time of the study. Despite the data being public, we took an additional step to protect the identity of the subscribers whose posts were included in the analysis by removing their subscriber handles before passing the data on to the annotators.

### Data Collection and Preparation

We collected data via the Twitter academic application programming interface (API) using monkeypox-related keywords and hashtags (eg, “monkeypox,” “#monkeypox,” “#mpoxvirus,” and “monkey pox”). We used the relevant API query filter to only collect posts in the English language. For multiword expressions, individual words were first used for streaming data collection and then regular expression-based matching was performed to filter out posts that did not contain the exact multiword expression. We collected data from August to October 2022—a 3-month period. All tweets were preprocessed via natural language processing methods, and duplicates were removed. We randomly sampled the remaining unique posts for manual analysis. Multiple posts from the same Twitter subscriber were not included. For anonymity purposes, while manually analyzing the data, the researchers used the integer IDs for the tweet extracts to hide the Twitter handles of the original posters from the annotators.

### Manual Data Annotation and Analysis

The 3 authors of this manuscript first manually reviewed a sample of posts to prepare a guideline for further annotation or coding. Based on the guidelines, the authors or annotators coded the random sample of posts into topics*.* Following an initial round of coding of a subsample, the authors resolved disagreements via discussion and identified overarching themes. The more fine-grained coded topics were mapped onto the overarching themes. The rest of the sampled tweets were then manually categorized into the chosen topics and then into themes. In total, we identified 8 themes. Each tweet was assigned only 1 theme based on the most appropriate fit, as determined by the annotators. The categorizations were finalized via discussion after the entire sample was coded.

## Results

### Data

In total, we collected 128,615 posts within the specified time period. After preprocessing and removing duplicates, we were left with 101,433 posts. A total of 200 posts were sampled and chosen for manual categorization. The initial discussion included 50 posts. In the end, all 200 posts were revisited to ensure accurate categorization.

### Manual Categorizations

Table 1 presents the relative distribution of the 8 themes. The description of each theme along with examples is provided in Table 2. Table 1 shows that the most common themes were monkeypox doubts and media (44/200, 22%); the former included posts that questioned the validity and seriousness of the disease, and the latter included news articles shared by Twitter subscribers; 19% (38/200) of the posts discussed how the virus was transmitted, including speculations that may or may not have been accurate, and 13% (25/200) of the posts represented knowledge shared about monkeypox, such as symptoms. Politics (18/200, 9%), monkeypox vaccine (16/200, 8%), effect of monkeypox (12/200, 6%), and general comment (3/200, 1%) were the rest of the identified themes, occurring less than 10% in the sample. Note that some posts included information that could be put into multiple themes; in our final discussion, we adjudicated the most appropriate theme label for each post in such cases.

**Table 1:** Relative distribution of manually identified themes in our reviewed sample (N=200).

Themes	Posts on Twitter, n (%)
Monkeypox doubts	44 (22)
Media	44 (22)
Transmission	38 (19)
Knowledge of monkeypox	25 (13)
Politics	18 (9)
Monkeypox vaccine	16 (8)
Effect of monkeypox	12 (6)
General comment	3 (1)

**Table 2:** All monkeypox-related themes, their descriptions, and examples (some examples have been shortened or anonymized).

Theme	Description of themes	Example
Monkeypox doubts	Doubts about the existence of monkeypox	*y’all remember #monkeypox ? what happened to it? not a thing any more?? people woke up to the silliness of it all??*
Media	Published and announced news	*monkeypox case rates 5 times higher in black americans* [link] *from @USERID*
Monkeypox transmission	Tweets suggesting mode of spread and risk factors	*Monkeypox spreads by close contact with someone who has symptoms or by touching contaminated items.*
Knowledge of monkeypox	Facts or information about monkeypox	*Monkeypox symptoms are mild, and the disease is rarely fatal. Everyone can help prevent the spread of Monkeypox.*
Politics	Comments related to political events	*monkeypox is not over & here to stay in the usa because of joe biden and his lack of response, americans aren’t getting their covid boosters because he said the pandemic is over again like he did a year and a half ago before the fourth of july and now these super bugs are coming*
Monkeypox vaccine	Views and information about monkeypox vaccine	*D.C. health officials have vaccinated people against monkeypox at a rate at least 56 times higher than maryland, according to a data analysis by the baltimore banner*
Effect of monkeypox	Consequences of being infected with monkeypox and the outbreak	*monkeypox caused encephalitis which caused death.*
General comment	General posts, posts lacking context, or posts unrelated to monkeypox	*there have* [sic] *been a consistently unreliable source of monkeypox information*

## Discussion

### Principal Results

Our study demonstrated that there was a substantial amount of monkeypox-related information on Twitter during the outbreak. This finding is unsurprising since much of the infodemiology research during and prior to COVID-19 presented similar findings. Our work adds further support to a growing body of research focusing on the intersection of social media and infectious diseases; it also supports the possibility of leveraging knowledge from social media to obtain early insights into infectious diseases and outbreaks.

In terms of the contents of the posts and their themes, we found a mixture of relevant and irrelevant or inaccurate information. Many posts expressed doubts or promoted conspiracy theories about the existence and origin of monkeypox. Posts also expressed political alignment, demonstrating how regional politics can shape people’s perceptions of the disease. This is perhaps the new normal in a post–COVID-19 world, where the spread of misinformation is inevitable. The presence of misinformation makes it even more important to conduct social media–based surveillance so that it can be detected early and public health agencies across the world can take the necessary steps to combat the issue.

Our review also found important information, such as symptoms, knowledge, and emerging news on monkeypox. Such information can be of particularly high utility at the early stages of infectious disease outbreaks when little is known about the topic. This utility was observed during the early stages of COVID-19 when social media chatter revealed symptoms associated with infection [9]. Studies, including ours, on COVID-19 and monkeypox have consistently found that social media subscribers share their personal experiences once infected, and there is the potential to conduct syndromic surveillance using such data. Many posts also quelled misinformation (eg, about how monkeypox spreads) posted by others.

### Comparison With Prior Work

About 40,000 monkeypox-related tweets were posted on the day monkeypox was declared a global public health emergency by the World Health Organization [8]. An early sentiment analysis of the monkeypox tweets revealed a larger number of neutral tweets, followed by negative and then positive tweets [8]. Most of the data revolved around Twitter subscribers’ discussions related to monkeypox. In another study, “expressed fear” or negative sentiments were the most frequent observations, followed by neutral or positive sentiments [10]. Fear and sadness were the most common emotions in negative sentiment Tweets. In addition to sharing their concerns and opinions, Twitter subscribers reportedly spread a lot of mis- and disinformation about monkeypox [11]. In a separate study also involving Twitter, the authors applied unsupervised learning, namely topic modeling, to identify topics related to monkeypox from a large number of posts [12]. Our work, in contrast, focused on a thorough manual categorization, so that topics and themes that are most relevant for subject matter experts were identified and coded. Besides Twitter, other social networks have also been leveraged to study monkeypox. In a study [13], 100 videos were analyzed; 55% of them were from health care professionals, 38% were from non–health care professionals, and 7% were from news organizations. The study found that while health care–related content was more accurate and of higher quality, viewers engaged more with the non–physician-created content [13].

### Limitations

Our study has several limitations. First, only a small sample of data was manually analyzed. Although we could have applied automated strategies, such as topic modeling, for analyzing the data, we intentionally chose to conduct a manual thematic analysis since our past research [14-16] has shown that such analyses provide more fine-grained and detailed insights. Second, we used a limited set of keywords for data collection and did not include lexical variants, potentially reducing the total number of posts. The general limitations of social media also apply to our work—social media subscribers are younger, on average, compared to the overall population, and they also tend to be more tech-savvy. People without access to the internet are automatically excluded from social media studies. Finally, our data collection period was short, due to the formative nature of this study.

### Conclusions

Our study revealed the major categories of discussion regarding monkeypox on Twitter. The findings shed light on the possible distribution of social media chatter associated with the most important manually identified themes. Unlike prior work that leveraged automated analyses, our work focused on identifying pertinent themes manually. This is a particular strength of this study since automated methods may not incorporate prior knowledge about the subject matter when identifying topics or themes that are the most relevant. Future work may use manual characterization to train supervised learning models for large-scale, automated analyses. Although our use case was monkeypox, our study suggests that social media data analysis can reveal important information about public perceptions in the early stages of infectious disease outbreaks. Social media data may also enable the early detection of misinformation regarding disease outbreaks, allowing public health agencies and policy makers to act accordingly.

## Abbreviations

API: application programming interface

## Data Availability

Since some of the opinions expressed in the posts can be controversial and some posts may self-disclose personal information, the data for this study have not been made publicly available to preserve the anonymity of the subscribers whose posts were analyzed. The study can be replicated by collecting publicly available Twitter posts via the appropriate application programming interface (API).
